# Why Canadians deserve to have mandated health and standard drink information labels on alcohol containers

**DOI:** 10.17269/s41997-023-00786-1

**Published:** 2023-06-19

**Authors:** Norman Giesbrecht, Ashley Wettlaufer, Kate Vallance, Erin Hobin, Timothy Naimi, Tina Price, Tim Stockwell

**Affiliations:** 1 Institute for Mental Health Policy Research, Centre for Addiction and Mental Health, Toronto, Ontario Canada; 2 Dalla Lana School of Public Health, University of Toronto, Toronto, Ontario Canada; 3 Canadian Institute for Substance Use Research, University of Victoria, Victoria, British Columbia Canada; 4 Public Health Ontario, Toronto, Ontario Canada

**Keywords:** Alcohol, Health information and warnings, Alcohol containers, Standard drinks, Myths, Alcool, indications et mises en garde sur la santé, contenants d’alcool, verres standards, mythes

## Abstract

To reduce deaths, morbidity, and social problems from alcohol in Canada, a multi-dimensional robust response is needed, including a comprehensive alcohol control strategy at the provincial, territorial, and federal levels. Alcohol container labels with health and standard drink information are an essential component of this strategy. This commentary provides a rationale for the mandatory labelling of all alcohol products, summarizes Canadian initiatives to date to legislate alcohol container warning labels, and addresses myths and misconceptions about labels. Canadians deserve direct, accessible information about (1) the inherent health risks associated with alcohol consumption, (2) the number of standard drinks per container and volume of a standard drink, and (3) guidance for preventing or reducing consumption-related health risks. Enhanced health labels on alcohol containers are long overdue.

## Résumé

Pour réduire les décès, la morbidité et les problèmes sociaux causés par l’alcool au Canada, il est nécessaire de réagir vigoureusement et de manière multidimensionnelle, notamment avec une stratégie globale de mesures de contrôle de la part des gouvernements provinciaux, territoriaux et fédéral. Des étiquettes sur les contenants d’alcool comportant des informations sur la santé et sur ce qui constitue un verre standard sont un élément important et essentiel de cette stratégie. Cette analyse explique les raisons justifiant l’étiquetage obligatoire de tous les produits alcoolisés, résume les initiatives à ce jour pour établir des lois sur les étiquettes de mise en garde sur les contenants d’alcool et réfute les mythes et les idées fausses sur ces étiquettes. Les Canadiens ont le droit d’être informés directement et de manière accessible sur 1) les risques pour leur santé inhérents à la consommation d’alcool, 2) le nombre de verres standards par contenant, et le volume d’un verre standard, et 3) les recommandations pour prévenir et réduire les risques liés à cette consommation. Nous n’avons que trop attendu pour apposer des étiquettes de mise en garde sur la santé sur les contenants d’alcool.

Alcohol is a dangerous drug. Roughly 17,000 Canadians die annually from alcohol-attributable causes, with many more involved in alcohol-attributable hospital stays or emergency room visits (Canadian Substance Use Costs & Harms Scientific Working Group, 2023). Alcohol consumption is associated with numerous chronic diseases, including seven types of cancer. Alcohol is also a factor in various types of trauma and social problems, including fetal alcohol effects, impaired driving crashes, and violence. Many victims are not drinkers nor high-risk drinkers (World Health Organization, 2018).

However, vital information about health risks of alcohol, the number of standard drinks per container, and national drinking guidance are not mandated for labels on alcohol products sold in Canada. By contrast, there are federally mandated warning labels on tobacco and cannabis packages.

People in Canada deserve direct and accessible information about the risks of alcohol. Three label messages should be included on alcohol containers to effectively support consumers in making more informed decisions: (1) the health and safety risks associated with alcohol consumption, (2) the number of Canadian standard drinks in the container and the volume of a standard drink, and (3) alcohol and health guidance for preventing or reducing consumption-related risks.

In this commentary, we provide a rationale for enhanced labels on alcohol containers. Also, we offer insights and evidence on why alcohol warning labels are not currently mandated on all alcohol beverages in Canada, and we refer to international experiences on this topic. We outline the components of proposed enhanced warning labels (Table 1) and provide examples of standard drinks using various alcohol beverage types (Table 2). We also highlight myths and misconceptions about alcohol warning labels and offer rebuttals (Table 3). Finally, we propose recommendations about how to move forward and address opposition from interests who oppose informing people in Canada about the harms from alcohol.

**Table 1:** Message components of an enhanced alcohol warning label

Component	Comment
Health warnings	Rotating messages referring to chronic diseases, including cancer; motor vehicle incidents; violence; FASD; alcohol dependence
Full-colour pictogram or image	
Standard drink information	How many standard drinks are in the container, what is the volume of a standard drink of ethanol (see Table 2)
Safer consumption guidance	Canadian Guidance on Alcohol and Health

Sources: Hobin et al., 2020; Kokole et al., 2021; World Health Organization, 2022; Giesbrecht et al., 2022; Paradis et al., 2023

*FASD* Fetal alcohol spectrum disorder

**Table 2:** Standard drinks in Canada

Beverage	Beverage volume	Ethanol strength (% alcohol by volume)	Ethanol volume	Standard drinks
Beer	341 ml (12 oz)	5%	17.05 ml	1.0
Pint	568 ml (20 oz)	5%	28.4 ml	1.66
Regular size table wine	148 ml (5 oz)	12%	17.76 ml	1.0
Larger size table wine	266 ml (9 oz)	12%	31.92 ml	1.79
One shot of spirits	44 ml (1.5 oz)	40%	17.6 ml	1.0
Double shot of spirits	88 ml (3.0 oz)	40%	35.2 ml	2.0

**Table 3:** Myths/misconceptions and rebuttals

Myths and misconceptions	Rebuttal
1. Labels don’t work	Increasing evidence suggests well-designed alcohol labels can be effective. Studies of US alcohol warning labels (AWLs) showed that the labels were more likely to be noticed by high-risk drinkers, impaired drivers, and pregnant women; labels also stimulated conversations about alcohol (Greenfield, 1997). The short-lived but successful Yukon experiment demonstrated increased awareness, knowledge of cancer risks, and reductions in consumption associated with AWLs (Hobin et al., 2020; Zhao et al., 2020). Furthermore, consumers have a right to know about hazards of the products they are using, regardless of labels’ direct impacts on consumption (Stockwell et al., 2020)
2. Science doesn’t support the health claims of alcohol and cancer	World Health Organization’s International Agency on Research in Cancer (IARC) monographs on alcohol and cancer (IARC, 2010) show there is very strong scientific evidence about alcohol-cancer relationships, established over a number of decades and which continues to grow (World Health Organization, 2018). Alcohol has long been known as a carcinogen with strong causal evidence for seven types of cancer, including cancers of the oropharynx, larynx, esophagus, liver, colon, rectum, and breast. In fact, alcohol is designated as a class 1 (most severe) carcinogen, along with tobacco smoke, ultraviolet rays, asbestos, outdoor air pollution, and benzene; IARC reserves this designation for substances with (1) strong epidemiological evidence in human studies, (2) strong experimental evidence from animal studies, and (3) biochemical evidence from laboratory and in vitro studies (IARC, 2010)
3. The information is too complex to fit on a label and would be best if provided on a website linked to a QR code at the point of purchase or via education campaigns	Evidence shows that information on health risks, standard drinks, and drinking guidelines is most effective when provided at the point of purchase and points when alcohol is poured and consumed, and that complementary education campaigns should be supplementary to labels (National Alcohol Strategy Advisory Committee, 2015). QR codes should not be used in place of labeling; the vast majority of consumers do not read QR codes (Budenz et al., 2022)
4. People already know not to drink while pregnant; putting it on the label further stigmatizes women who choose to drink	Not all persons are aware of the fact that alcohol can cause prenatal brain damage, congenital anomalies, growth restrictions, and cognitive deficits; estimates of alcohol use in pregnancy remain high (Popova et al., 2017). Providing information about the risks of drinking in and around pregnancy is not stigmatizing to women, but rather promotes autonomy in informed decision-making
5. Changing labels and providing coloured warnings and health information are cost prohibitive for manufacturers	Alcohol manufacturers regularly change label content and use a variety of colours and messaging for marketing purposes (e.g., rainbow packaging for Pride month, or holiday-themed packages), and already adhere to other label requirements. Further, other countries such as Australia and New Zealand (Food Standards Australia & New Zealand, 2022) require enhanced label components including standard drink information and a pregnancy warning, and Ireland will soon have mandated pregnancy and cancer warnings (Irish Statute Book, 2018)
6. There is no such thing as a standard drink, it’s a confusing concept for consumers to understand, and may contradict other label components, e.g., beer common names (strong beer, beer, light beer, and extra light beer)	The concept of a standard drink (SD) is an internationally recognized way of measuring the ethanol content in different types and strengths of beverages (see Table 2 of this paper). The wide range of beverage strengths and serving sizes across different types of alcohol products and even within beverage categories (e.g., varieties of wine) is precisely why standard drink information is crucial for informing consumers
7. People will use standard drinks information to select the products that give them the most bang for their buck	Studies show this isn’t the case; respondents are more likely to use the standard drink labels to follow drinking guidelines or to adhere to medical advice (Schoueri-Mychasiw et al., 2020)
8. Alcohol is beneficial for health so not equal to other beverages	The World Heart Federation (2022) now disputes the evidence of heart benefits from moderate drinking, including wine. The drinking guidance on labels is designed to protect health and prevent harms
9. Many people don’t see the container when they are drinking so enhanced alcohol labels will be useless	Those who use alcohol and the people around them will inevitably see alcohol containers at some point, whether in a liquor store, a restaurant, or in the fridge, and a substantial proportion of alcohol consumed is from single-serve containers
10. Consumers don’t need to be told what to do	Providing adequate, accessible, and transparent information allows people to make their own informed judgements and fulfils the manufacturers’ legal duty to inform consumers of the risks associated with their products. The lack of standard drink labelling and alcohol guideline information on alcohol containers is akin to having no speedometer in your car and no posted speed limits on the road. Further, given that an estimated two in five Canadians will have a cancer diagnosis in their lifetime (Public Health Agency of Canada, 2021), consumers deserve to know that alcohol consumption is a modifiable risk factor for a serious and prevalent health condition
11. Different types of alcohol (beer, wine, spirits) are more/less harmful and/or processed differently by the body (e.g., depending on whether they are carbonated or higher/lower alcohol content) and hence standard drinks are not equal	Ethanol is what causes harms and is what is contained in all types of alcoholic beverages, and epidemiological evidence shows that mortality risk is due to total alcohol consumed regardless of beverage type (WHO 2018)
12. Different people react very differently to alcohol and so it makes no sense to standardize the amount of alcohol for something that has wildly different effects on people	For each consumer, one standard drink has a generally predictable effect. Access to standard drink information allows consumers to calibrate the number of drinks to their effects

## Rationale for enhanced labels on alcohol containers

First, alcohol is deserving of specific attention because of its widespread use and status as Canada’s most popular legal drug. Second, alcohol is associated with a number of social, chronic, and acute health risks and potential harms, many of which are not well known or are poorly understood by the general public. Consumers have a right to know about hazards of the products they are using, regardless of labels’ direct impacts on consumption (Stockwell et al., 2020; World Health Organization, 2022). Third, there is good evidence that labels on alcohol containers can increase awareness of alcohol-related health effects (Greenfield, 1997; Hobin et al., 2020) and can change behaviour (Zhao et al., 2020). Warning labels can reach the majority of consumers, and are seen more often by more frequent consumers (Greenfield, 1997). Fourth, enhanced alcohol labels may offer support for other alcohol strategies and interventions by presenting an indirect rationale for such policies and reinforcing the importance of implementing a comprehensive strategy. For example, Canadian evidence indicates that enhanced labels facilitated knowledge about the link between alcohol and cancer and increased the likelihood of support for alcohol pricing policies (Weerasinghe et al., 2020). And fifth, in our view and that of legal scholars, Canadian manufacturers and suppliers have a legal duty to inform consumers of the risk inherent in the foreseeable use and misuse of their products (Stockwell et al., 2020). This duty to inform consumers extends to the federal, provincial, and territorial governments, given their responsibility to act in the public interest and roles as regulators in all provinces and territories, as well as to distributors of alcohol through government wholesale and retail operations.

## Why there are not mandated alcohol warning labels in all Canadian jurisdictions

To date, the Canadian experience with regard to implementing alcohol warning labels has been frustrating. The earlier experience is summarized in a case study by Ogborne et al. (2006). Between 1988 and 1991, four private members bills were presented to the federal Parliament under a Progressive Conservative Government that did not go to the committee stage. In 1995, a bill by Paul Zabo, Liberal backbencher, reached second reading and went to committee. However, in May 1996, Liberal Health Minister David Dingwall opposed this Bill C-222 (35–2), indicating there was no evidence of warning-label effectiveness, and stating that he had been advised by the alcohol industry it would cost $10 million to change the labels (Ogborne et al., 2006).

Despite the support for labels from Fetal Alcohol Spectrum Disorder (FASD) prevention groups, Mothers Against Drunk Driving (Canada), and other organizations, these legislative initiatives did not succeed. The alcohol industry lobbied MPs, claiming labels were ineffective, based on a selective assessment of the United States label’s experience. The industry also claimed high cost and threatened to withdraw funding for other educational programs if labels were introduced (Ogborne et al., 2006). In 2007, there was another attempt to have mandated labels which did not succeed (House of Commons, Bill C-251 n.d.).

There are currently two Canadian jurisdictions, Yukon and Northwest Territories, which have applied post-manufacturer labels on alcohol containers by local directive since 1991. Label messages include a pregnancy warning (Yukon) and a pregnancy, impaired driving, and general health warning (Northwest Territories).

In 2017, Erin Hobin and Tim Stockwell (two authors of this commentary) initiated a real-world study in Yukon and Northwest Territories using a quasi-experimental design to assess whether and how enhanced warning labels impact consumer perceptions, intentions, and behaviour. In the experimental site in Whitehorse, one of three new warning labels referred to alcohol as a carcinogen, namely: “Chief Medical Officer of Health advises Alcohol can cause cancer Including breast and colon cancers”. The alcohol industry threatened the Yukon Government with legal action and the labels referring to cancer were removed from rotation one month into the study. Legal scholars found that the industry objections were without merit (Stockwell et al., 2020); nevertheless, the research project was impacted by the interference. While there may be other factors which contribute to the absence of mandated alcohol warning labels in Canada, the resistance of the alcohol industry and their success in intimidating and influencing various governmental entities appear to be the pre-eminent explanations.

## International comparisons

Alcohol warning labels are required in several countries, although many have not been evaluated for their impact on perceptions, awareness, or behaviour (Giesbrecht et al., 2022). In 43 countries in the Americas, there are warning labels, with about equal number being mandated or voluntary. With exception of the US label introduced in 1989 that refers to multiple risks, these tend to be simple messages such as referring generally to health harm or addiction, or involving icons related to drinking during pregnancy, drinking and driving, or legal age limit (Giesbrecht et al., 2022). The 10 countries of the Commonwealth of Independent States (former USSR) have mandated labels; however, their impact has not been assessed (Neufeld et al., 2020). It is noteworthy that the US labels were more likely to be noticed by high-risk drinkers, impaired drivers, and pregnant women; the labels also stimulated conversations about alcohol (Greenfield, 1997).

Countries such as Australia and New Zealand (Food Standards Australia & New Zealand, 2022) require enhanced label components on alcohol containers, including standard drink information and a pregnancy warning. Ireland will soon implement mandated pregnancy and cancer warnings (Irish Statute Book, 2018). Canada now has the unique opportunity to become a world leader by implementing mandated enhanced health warning labels on alcohol containers, with a design that is based on recent evidence.

A bill currently in the Canadian Senate (B-S254) and a motion (M-61) put forward in the House of Commons speak to renewed public interest in this issue, yet the potential for industry resistance and interference remains, including via promotion of myths and misconceptions about alcohol labels (Table 3).

## Recommendations

Achieving mandated enhanced labels on alcohol products in Canada will require strong advocacy and public support. In order to counter common myths and misconceptions about warning labels (Table 3) and other issues raised by commercially vested interests, a robust and multi-dimensional response is strongly advised. Key strategies might include letter-writing and advocacy campaigns, contacting members of parliament and seeking meetings with decision-makers, and coalition building. These communications should highlight the harms from alcohol, benefits of alcohol warning labels, and the perceived legal duty to advise Canadians of alcohol-related risks. Working with other groups committed to reducing alcohol-related harm demonstrates multi-sectoral support for warning labels. Such groups include public health associations and departments, and organizations focused on preventing and treating FASD, preventing cancer, curtailing alcohol-related violence, and reducing impaired driving. The response will also benefit from a strong media-advocacy strategy which will help bring the issues to the attention of people in Canada. Demonstrating the range and strength of support for an evidence-informed approach to warning Canadians about alcohol-related harms will make it easier for decision-makers to take policy action on this issue.

## Conclusion

The Federal Government of Canada is already in a position to use its existing authority under the Food and Drugs Act (FDA) to mandate alcohol labels with health warnings, national drinking guidance, and standard drink information to raise awareness among alcohol consumers, potential consumers, and the broader public about the risks associated with alcohol use. Taking such action would be in keeping with the recent call for labels that formed part of the new Canadian Guidance on Alcohol and Health (Paradis et al., 2023).

## Data Availability

Not applicable.
